# Dosimetric consequences of rotational setup errors with direct simulation in a treatment planning system for fractionated stereotactic radiotherapy

**DOI:** 10.1120/jacmp.v12i3.3422

**Published:** 2011-04-04

**Authors:** Jean L. Peng, Chihray Liu, Yu Chen, Robert J. Amdur, Kenneth Vanek, Jonathan G. Li

**Affiliations:** ^1^ Department of Radiation Oncology Medical University of South Carolina Charleston South Carolina 29425; ^2^ Department of Radiation Oncology University of Florida Gainesville Florida 32610‐0385; ^3^ TomoTherapy, Inc. Madison WI 53717‐1954 USA

**Keywords:** fractional stereotactic radiotherapy (SRT), rotational positioning errors, dosimetry effects, direct simulation, treatment planning system (TPS)

## Abstract

The purpose was to determine dose‐delivery errors resulting from systematic rotational setup errors for fractionated stereotactic radiotherapy using direct simulation in a treatment planning system. Ten patients with brain tumors who received intensity‐modulated radiotherapy had dose distributions re‐evaluated to assess the impact of systematic rotational setup errors. The dosimetric effect of rotational setup errors was simulated by rotating images and contours using a 3 by 3 rotational matrix. Combined rotational errors of ± 1°,± 3°,± 5° and ± 7° and residual translation errors of 1 mm along each axis were simulated. Dosimetric effects of the rotated images were evaluated by recomputing dose distributions and compared with the original plan. The mean volume of CTV that received the prescription dose decreased from 99.3%± 0.5% (original) to 98.6%± 1.6% (± 1°), 97.0%± 2.0% (± 3°), 93.1%± 4.6% (± 5°), and 87.8%± 14.2% (± 7°). Minimal changes in the cold and hot spots were seen in the CTV. In general, the increase in the volumes of the organs at risk (OARs) receiving the tolerance doses was small and did not exceed the tolerance, except for cases where the OARs were in close proximity to the PTV. For intracranial tumors treated with IMRT with a CTV‐to‐PTV margin of 3 mm, rotational setup errors of 3° or less didn't decrease the CTV coverage to less than 95% in most cases. However, for large targets with irregular or elliptical shapes, the target coverage decreased significantly as rotational errors of 5° or more were present. Our results indicate that setup margins are warranted even in the absence of translational setup errors to account for rotational setup errors. Rotational setup errors should be evaluated carefully for clinical cases involving large tumor sizes and for targets with elliptical or irregular shape, as well as when isocenter is away from the center of the PTV or OARs are in close proximity to the target volumes.

PACS number: 87.53.Bn

## I. INTRODUCTION

Technical advances in radiotherapy over the last few years have made it possible to devise highly conformal treatment plans to the target volume and spare adjacent critical structures. However, the efficacy of radiotherapy can be compromised by errors in the treatment setup of the patient which affects the delivered radiation. With intensity‐modulated radiotherapy (IMRT) creating steep dose gradients and tight margins around the tumor, there is a greater risk of dose to surrounding organs at risk (OARs) due to setup errors and organ motion. Image‐guided radiation therapy (IGRT), whereby imaging devices are used at the time of treatment delivery to increase the probability that radiation is delivered as closely as possible to the original plan,^(1)^ can help avoid these problems. The practice of imaging treatment fields to localize the tumor has been carried out for several decades and has been referred to as megavoltage (MV) portal imaging. MV portal imaging has been performed using two‐dimensional (2D) X‐ray detectors to verify orthogonal localization fields and treatment ports. In most situations, only bony structures are used to assess the setup deviations in 2D portal images because soft tissues are difficult to visualize in the planar‐projection images.^(2)^ Computed tomography (CT) images are capable of identifying both bony structures and soft tissues, and CT is the standard reference to delineate organs and the target in treatment planning systems (TPSs). It is not surprising that volumetric CT registration in six degrees of freedom (DOF) performs more accurately than bony‐structure registration based on 2D portal images.^(2)^ There is a growing interest in CT‐based imaging systems for 3D volumetric localization.

The most common of the sophisticated image‐guided approaches is the gantry‐mounted cone‐beam CT (CBCT) systems because of its complete set of 3D volumetric information with patient in the treatment position.^(^ ^2^ ^–^ ^6^ ^)^ It has been shown that the translational and rotational setup errors can be correctly determined with six DOF registrations using the CBCT systems, making them suitable for high‐precision treatments like stereotactic radiosurgery.^(^ ^4^ ^,^ ^5^ ^)^ Although conventional treatment tables do not allow rotational corrections, six DOF corrections can be achieved with a robotic couch,^(^ ^7^ ^,^ ^8^ ^)^ or by using a combination of collimator, gantry, and/or couch rotations.^(^ ^9^ ^,^ ^10^ ^,^ ^11^ ^)^ Strategies for positioning corrections when rotational errors are not adjusted have also been discussed.^(12)^ The post or near real‐time verification^(^ ^7^ ^,^ ^8^ ^)^ after corrections is essential. Currently, most setup adjustments are applied to the translational direction only, and rotational positioning errors still exist throughout the patient's treatment.

Mathematically, a 2° rotational deviation induces a maximum translational deviation of about 1 mm at a point located 3 cm from the isocenter. For elongated targets (> 5cm long), with rotational deviations greater than 2°, the translational deviation is more than 1.8 mm, which could be significant when a tight margin is used.^(^ ^3^ ^,^ ^13^ ^)^ Guckenberger et al.^(3)^ found that the maximal rotational errors using kV CBCT were 5°, 8°, and 6° for pelvic, thoracic, and head and neck tumors, respectively. Based on daily MVCT from 3,800 tomotherapy treatments,^(6)^ at least 5% of brain patients had more than 3° in roll rotations. Those rotational positioning errors resulted in decreased target coverage and increased dose to the OARs. However, many factors contribute to the dosimetric effect, including the target size, geometric relationship between the target, OARs, and beam arrangement, degree of dose‐gradient steepness, margin sizes, and treatment techniques. The quantitative correlation between the amount of rotational errors and their dosimetric consequence is not obvious and there is a wide range of dose effects.^(^ ^3^ ^,^ ^13^ ^–^ ^18^ ^)^ For example, during simulation, the simplest approach is to rotate the gantry for roll correction and couch for yaw correction.^(^ ^13^ ^,^ ^16^ ^,^ ^8^ ^)^ Yue et al.^(9)^ developed a method to implement all six DOF corrections using combinations of gantry, collimator, and couch rotations achievable with a conventional treatment couch.^(^ ^14^ ^,^ ^15^ ^)^ Additionally, for online corrections, both patient repositioning and plan adjustments have been proposed, and CBCT images have been used to evaluate dose impacts on setup errors.^(^ ^3^ ^,^ ^17^ ^)^ At present, it is still a challenging task to accurately delineate the tumor and organs, and calculate the dose using CBCT images.^(19)^

At the University of Florida, a frameless biteplate system is used for patient setup and localization for fractionated stereotactic radiotherapy (SRT) of brain tumors.^(20)^ Patient setup is verified using CBCT for the first three fractions and then on a weekly basis. The biteplate system monitors patient positioning in real time and provides consistent inter‐fractional patient setup with submillimeter precision. However, systematic differences occur when verified using CBCT. Such differences could arise from calibration differences of the two systems or systematic patient bite differences between simulation and treatment. While translational errors can be corrected with a simple couch shift, rotational errors cannot be easily accounted for and therefore exist throughout the course of treatment. Such rotational errors represent a systematic difference between patient simulation and treatment, and the dosimetric consequences need to be carefully evaluated.

The purpose of this study is to use direct simulation in TPS to evaluate the dosimetric consequence of systematic rotational setup errors in fractionated SRT treated with IMRT. The simple and direct simulation method was achieved by rotating the original CT image set and the associated contours of targets and OARs. We examined the accuracy of this method by registering the image sets and calculating the volume differences of the contoured structures. All dose calculations and comparison were done in the TPS by using those rotated image sets and contours. For a group of SRT patients, the simulated dose distributions with rotational errors were retrospectively calculated and compared with the original plans using dose‐volume histogram (DVH) parameters to investigate the dosimetric effects.

## II. MATERIALS AND METHODS

### A. Patient selection

**Figure 1:**
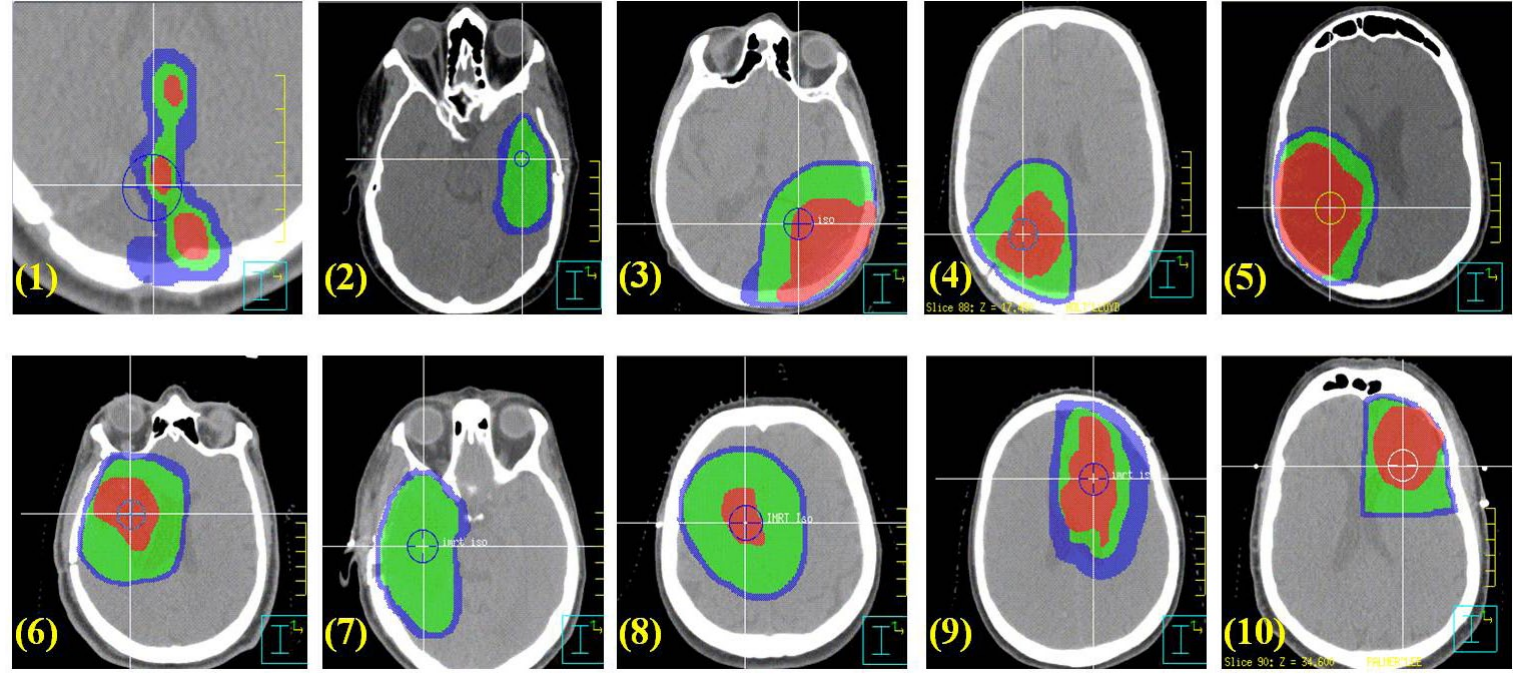
Tumor sites and shapes for the ten cases in this study: GTV (red): gross tumor volume; CTV (green): clinical target volume; PTV (blue): planning target volume. The numbers correspond to the case numbers in Table 1. The circles represent the isocenters.

Ten SRT patients with intracranial tumors who had undergone IMRT between August 2008 and April 2009 in our institution were selected for the study. We selected patients whose targets were located at different areas with different sizes and shapes in order to study the dosimetric impacts of rotational setup errors in these situations. Table 1 summarizes the patient information and the characteristics of the target volumes. Histologies and staging were determined by clinical evaluation. Figure 1 displays the cropped CT images of the tumors for each case. As shown in Figure 1, the shape and size of the targets varied considerably from patient to patient. The target shapes were classified into three categories: sphere, ellipse, and irregular. All glioblastmoas were shaped regularly or elliptically. The base‐of‐skull meningioma was irregularly shaped, mostly because of extensions in the cavernous sinus and through foramina. The mean PTV volume and largest dimension were 232.5 cm^3^ (range: 90.4 cm^3^ – 376.5 cm^3^) and 9.3 cm (range: 7.2 cm–12.1 cm), respectively, to study the effect of the target size and shape.

**Table 1:** Summary of characteristics of the 10 cases in this study.

			*PTV*			
*Case* #	*Diagnosis*	*Site*	*Volume* $\left({\text{cm}}^{3}\right)$	*Shape*	*Largest Dimension (cm)*	*OAR Considered*
1	Meningioma	R. Occipital	97.8	Irregular	7.7	Brain Stem, Chiasm, Retina, Lens, Opt. N
2	Glioblastoma	L. Temporal	90.4	Ellipse	7.8	Brain Stem
3	Glioblastoma	L. Parietal	370.6	Ellipse	12.1	Brain Stem, Chiasm, Retina, Lens, Opt. N, Cochlea
4	Glioblastoma	R Parieto‐occipital	284.3	Sphere	9.5	Brain Stem
5	Astrocytoma	R. Parietal	312.0	Sphere	9.9	Brain Stem
6	Oligoendroglioma	R Temporal	376.5	Sphere	9.3	Brain Stem, Chiasm, Retina, Lens, Opt. N
7	Glioblastoma	R. Temporal	131.4	Ellipse	10.1	Brain Stem, Retina, Lens, Opt. N., Cochlea
8	Oligodendroglioma	R. Parietal	260.0	Sphere	8.6	Brain Stem, Chiasm, Retina, Lens, Opt. N.
9	Glioblastoma	L. Frontal	323.0	Ellipse	11.2	Brain Stem, Chiasm, Retina, Opt. N
10	Glioblastoma	L. Frontal	213.9	Sphere	7.2	Brain Stem, Chiasm, Retina, Lens, Opt. N

PTV: Planning target volume; OAR: organ at risk; Opt. N: optical nerves

### B. Simulation and target delineation

Each patient was immobilized with a custom disposable head support (MoldCare Pillow, Bionix Inc, Toledo, OH) combined with a half‐face thermal plastic (TP) mask. A bite block using teeth impression was custom‐made for localization using a camera system (SonArray, Varian Medical Systems, Palo Alto, CA). A CT scan was acquired using a multi‐slice CT scanner (Philips Medical Systems, Cleveland, OH) with a 2 mm slice thickness and $0.98\times 0.98\;{\text{mm}}^{2}$ in‐plane resolution. T1‐weighted magnetic resonance (MR) images were also acquired for all the cases to facilitate target volume definition. Coregistration of CT and MR images and all contour delineations were done using a commercial treatment planning system (Pinnacle^3^, ver 8.0m, Philips Medical Systems, Madison, WI). A uniform margin of 3 mm was initially used to create the PTV from the CTV. For patients in whom the CTV was in close contact with OARs, the PTV margin was reduced manually to avoid overlapping of the PTV and the OARs. OARs included brain stem, optical chiasm, optical nerves, retina, cochlea, and lens.

IMRT treatment plans were designed on the commercial TPS using four to nine non‐coplanar beams. Dose prescription in all cases was 61.2 Gy with 1.8 Gy per fraction. The goal of target coverage included ensuring that 95% of PTV received the prescription dose $\left(V_{100\text{Rx}}\geq 95\%\right)$, more than 99% of PTV received 93% of the prescription dose $\left(V_{93\text{Rx}}\geq 99\%\right)$, and no more than 20% of PTV received more than 110% of the prescription dose $\left(V_{110\text{Rx}}\leq 20\%\right)$, For OARs, the following dose parameters were evaluated and limited to $0.1\;{\text{cm}}^{3}:V_{55\;\text{Gy}}$ (volume receiving ≥ 55 Gy) for the brain stem, chiasm, and optical nerve; $V_{45\;\text{Gy}}$ for the retina and cochlea; and $V_{12\;\text{Gy}}$ for the lens. All dose calculations were done using the convolution/superposition algorithm with a dose grid size of 2×2×2 mm^3^.

### C. Simulation of patient rotation with respect to isocenter

**Figure 2:**
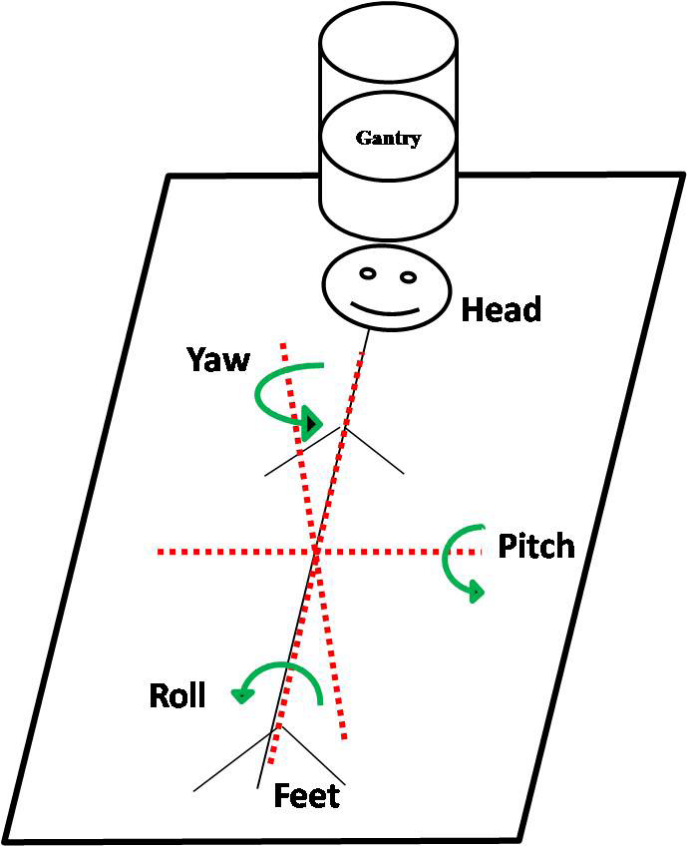
Coordinate system used in the study. Arrows indicate positive rotation with respect to each axis.

**Figure 3:**
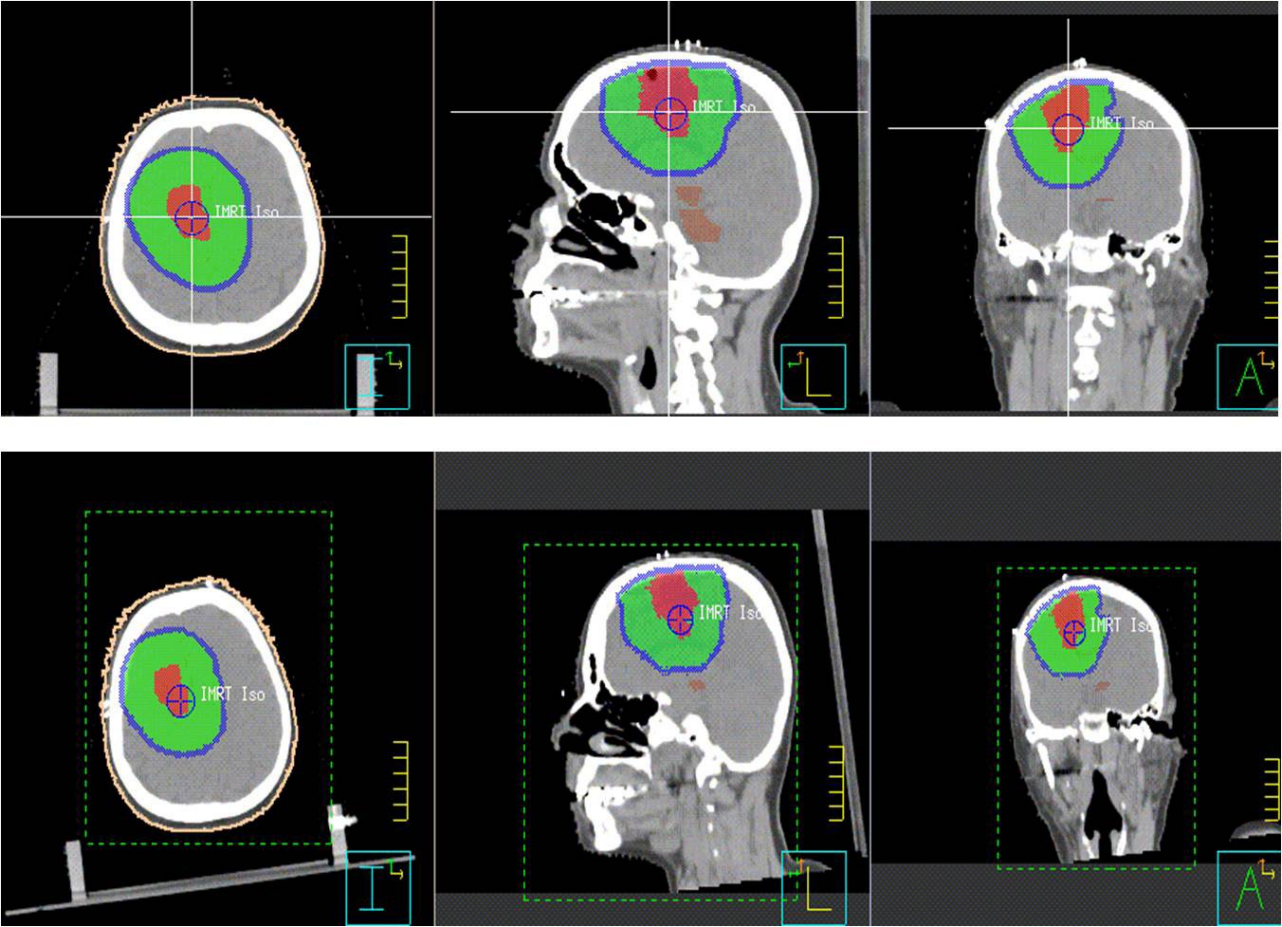
Example of image and structure rotation for case #8 with +7° rotation in three axes. The upper row shows the original images and target structures in the three orthogonal planes; the lower row shows the rotated images and target structures depicting rotational setup errors.

The dosimetric effect of systematic rotational setup errors on each patient's treatment plan was evaluated by simulating multiple rotational setup errors and recomputing the dose distributions in the TPS. Dose consequences were analyzed with the conservative assumption that systematic errors existed throughout the treatment. Since the commercial TPS did not provide the tools for image rotation and dose calculation on the rotated images, image and structure rotations and residual translations were simulated using a software program developed in MATLAB (2009b, The MathWorks, Natick, MA). The rotated images were imported back to the TPS for dose calculation and DVH analysis. The difference between the original image set [O] and the translated/rotated image set [$O_{R}$] can be completely represented by a 3×3 rotation matrix [*R*] and a translation vector *T*, as shown Eq. (1):

(1) $$[O_{R}]=[O][R]+T\ldots \;\ldots \;\ldots \;\ldots \;\ldots \;\ldots \;\ldots \;\ldots \;$$

where $\left[R]=[R_{\text{pitch}}][R_{\text{roll}}][R_{\text{yaw}}\right]$ represents the combination of rotations in pitch, roll, and yaw. Figure 2 shows the definition of pitch, roll, and yaw used in this study. All simulated rotations were with respect to the treatment isocenter. For each patient, ± 1°,± 3°,± 5°, and ± 7° rotations in three axes were simulated and combined with a 1 mm translation in the three axes, which were used to represent residual errors after CBCT corrections. Figure 3 shows an example of the original and rotated images with a rotation angle of +7° in three axes for case #8. For each case, the original plan was regenerated eight times using each rotation angle, and the same beams as the original plan were used for dose calculation. The resulting dose distributions represent the distributions with the rotational setup errors.

The accuracy of the image rotation algorithm was validated using the image registration package (Syntegra, Philips Medical Systems, Andover, MA) within the TPS. The translated and rotated images were registered with the original images and both the differences between the intended translation/rotation and the registration results and the differences between the structure volumes of the original and rotated images were compared. A total of 90 plans were analyzed for the ten patients (one original and eight rotated plans for each patient). The DVHs of the rotated plans for the CTV and six OARs were evaluated with respect to the planning objectives outlined above and with those obtained from the original plan.

## III. RESULTS

### A. Validation of simulated images and contours

The isocenter transformation of each image set after registering with the original image set is shown in Table 2. The mean translational and rotational errors were 0.3±0.4 mm and 0.1° ± 0.2° in any axis, respectively, and the maximum errors were 0.8 mm and 0.4° for all 80 rotated images (eight rotated images per patient). Table 3 shows the volume differences between the rotated and original structures in absolute and percentage values for all the targets and OARs. The maximum error in the absolute volume differences was 1.8 cm^3^, which happened in one of the largest PTVs (case #1). By comparison, the percent volume differences were approximately 0.5% for all the targets, and < 2% for OARs with volumes >1 cm^3^. The maximum percentage errors were < 5% when the volume was <1 cm^3^.

**Table 2:** Isocenter transforms of rotated CT image set after registration process with original CT image set.

	±1°/1 mm		±3°/1 mm		±5°/1 mm		±7°/1 mm	
*Rotated Image Sets*	*Trans. (mm)*	*Rots. (*°*)*	*Trans. (mm)*	*Rots. (*°*)*	*Trans. (mm)*	*Rots. (°)*	*Trans. (mm)*	*Rots. (°)*
LAT.	1.0±0.2	±1.0±0.1	1.0±0.2	±3.0±0.1	1.0±0.2	±5.1±0.1	1.0±0.2	±7.0±0.1
VERT.	1.0±0.1	±1.0±0.1	1.0±0.2	±3.1±0.2	1.0±0.1	±5.0±0.1	1.1±0.2	±6.9±0.2
LONG.	0.7±0.4	±1.0±0.1	0.8±0.3	±3.0±0.1	0.7±0.5	±5.0±0.0	0.7±0.3	±7.0±0.1

LAT.=lateral (left‐right); LONG.=longitudinal (superior‐inferior); VERT.=vertical (anterior‐posterior); Trans.=translations; Rots.=rotations.

**Table 3:** Absolute and percentage (%) differences in volume (V.) between the original and rotated structures.

*Structures*	*CTV*	*PTV*	*Brain Stem*	*Chiasm*	*Retina*	*Opt.N*	*Lens*	*Cochlea*
Mean V.	162.6±	232.5±	27.6±	0.6±	5.5±	1.0±	0.3±	0.08±
$\left({\text{cm}}^{3}\right)$	85.4	103.8	3.4	0.4	0.9	0.5	0.2	0.03
*Absolute Volume Difference* $\left({\text{cm}}^{3}\right)$								
±1°/1 mm	0.4± 0.5±	0.2±	0.01±	0.05±	0.02±	0.003±	0.0003±
	0.5	0.8	0.3	0.06	0.02	0.06	0.009	0.006
±3°/1 mm	0.6±	0.7±	0.2±	0.01±	0.08±	0.01±	0.009±	0.0004±
	0.8	1.1	0.3	0.06	0.09	0.07	0.03	0.006
±5°/1 mm	0.2±	0.7±	0.1±	0.01±	0.07±	0.02±	0.01±	0.0001±
	1.0	1.1	0.4	0.05	0.02	0.07	0.03	0.008
±7°/1 mm	0.3±	0.8±	0.3±	0.01±	0.08±	0.02±	0.01±	0.0004±
	1.4	1.5	0.3	0.05	0.07	0.06	0.04	0.003
% differences	~0.4%	~0.4%	~1.1%	~1.7%	~1.5%	~2.0%	~2.9%	~4.9%

Mean V. = mean volume of the original structures.

### B. Target dose assessments and comparisons

Table 4 summarizes the $V_{100\text{Rx}}$ and $V_{93\text{Rx}}$ for the CTV in the original (0°) and rotated (± 1° ~ ± 7°) plans for all the cases and the averaged values for each of the rotations. Significantly greater loss of coverage was seen in $V_{100\text{Rx}}$ than $V_{93\text{Rx}}$. A systematic rotation of 5° or more reduced the $V_{100\text{Rx}}$ to less than 95% in five of the ten cases. On the other hand, only one case (case #3) had $V_{100\text{Rx}}$ reduced to less than 95% with a 3° or less rotation. Systematic rotation errors caused minimal coverage loss (less than 3%) in $V_{93\text{Rx}}$ except in a few cases with rotational errors greater than 5°. Changes in $V_{110\text{Rx}}$ were all negligible (< 1%) in all cases.

**Table 4:** Effect of rotational setup errors on target coverage in IMRT for SRT (unit: %volume).

		$\text{CTV}V_{100\text{Rx}}(\%)$					$\text{CTV}V_{93\text{Rx}}(\%)$				
*Case #*	*PTV Shape*	*Original*	±1°	±3°	±5°	±7°	*Original*	±1°	±3°	±5°	±7°
1	Irregular	99.6	99.1	95.7	87.2	76.1	100.0	100.0	100.0	98.2	92.0
2	Ellipse	99.8	99.3	95.6	88.6	77.2	100.0	100.0	100.0	100.0	98.3
3	Ellipse	99.2	95.8	92.7	86.1	79.3	100.0	100.0	100.0	97.7	92.7
4	Sphere	99.8	99.1	96.8	93.8	86.9	100.0	100.0	100.0	100.0	99.7
5	Sphere	100.0	99.8	97.9	92.5	85.3	100.0	100.0	100.0	100.0	99.8
6	Sphere	99.0	98.9	98.4	95.9	96.2	100.0	100.0	100.0	99.9	100.0
7	Ellipse	98.4	97.8	97.6	96.4	93.0	100.0	99.9	100.0	100.0	99.9
8	Sphere	99.2	98.3	98.0	96.3	93.3	100.0	100.0	100.0	100.0	100.0
9	Ellipse	98.6	98.8	98.7	97.9	96.4	100.0	100.0	100.0	100.0	99.9
10	Sphere	99.0	99.0	98.2	96.7	94.1	100.0	100.0	100.0	100.0	99.0
Average	99.3±	98.6±	97.0±	93.1±	87.8±	100.0±	100.0±	100.0±	99.6±	98.1±
	0.5	1.6	2.0	4.6	14.2	0.1	0.0	0.0	0.6	4.7

$V_{100\text{Rx}},V_{93\text{Rx}}$ = percentage volume receiving 100% and 93% of prescribed dose, respectively.

### C. OAR dose assessments and comparisons

Table 5 summarizes the changes in volumes of the OARs receiving more than the tolerance doses in the rotated plans. In general, the increase in the volumes receiving the tolerance doses was small and did not exceed the tolerance for all rotated plans. This was achieved by the use of non‐coplanar beams which avoided beam entrance and exit through the OARs. The notable exception is the brain stem, where a significant increase in $V_{55\;\text{Gy}}$ was observed. Cases #6 and #7 experienced the most dramatic increase in $V_{55\;\text{Gy}}$, with an average increase of 0.9 cm^3^ with ±1°,2.0 cm^3^ with ±3°,2.8 cm^3^ with ± 5°, and 3.7 cm^3^ with ± 7°. Figure 1 indicates that for these two cases, the brain stems were in close proximity to the target volumes and, therefore, were located in or close to the high dose gradient areas. A small rotational error would necessarily have a significant dosimetric impact.

**Table 5:** The difference in volumes of OARs that received the tolerance dose between rotated and original plans.

	$V_{55\text{Gy}}$			$V_{45\text{Gy}}$		$V_{12\text{Gy}}$
	*Brain Stem*	*Chiasm*	*Opt.N*	*Retina*	*Cochlea*	*Lens*
Mean V.(cm^3^)	27.6±3.4	0.6±0.4	1.0±0.5	5.5±0.9	0.08±0.03	0.3±0.2
±1°	0.2±0.3	0.0±0.1	0.0±0.1	0.0±0.0	0.0±0.0	0.0±0.0
±3°	0.2±0.6	0.0±0.1	0.0±0.1	0.0±0.0	0.0±0.0	0.0±0.0
±5°	0.3±0.9	0.0±0.1	0.0±0.1	0.0±0.1	0.0±0.0	0.0±0.1
±7°	0.4±1.3	0.0±0.1	0.0±0.1	0.1±0.3	0.0±0.0	0.1±0.2

$V_{55\;\text{Gy}},V_{45\;\text{Gy}},V_{12\;\text{Gy}}$ = volume (cm^3^) receiving 55 Gy, 45 Gy and 12 Gy or more, respectively

## IV. DISCUSSION

For intracranial cancer patients treated with IMRT, our results demonstrated that dosimetric deviations increase with increases in rotational setup errors. For systematic rotational setup errors up to ± 3°, the probability of coverage loss to the CTV > 5% and of increased volumes receiving the tolerance dose to OARs >0.1 cm^3^ is small with a 3 mm CTV‐to‐PTV margin. The effect appeared to be associated with the relative location of the tumor and OARs, the shape and size of the tumor and OARs, and the relative location of the isocenter in the tumor‐OAR geometry. Larger targets and those with irregular shapes were more susceptible to rotational setup errors.^(14)^ For example, for case #3, the combination of larger tumor size, elliptical shape, and isocenter away from the center of the PTV contributed to the most consequential dosimetric impacts on CTV coverage, with $V_{100\text{Rx}}$ for CTV decreased by more than 3% with only ± 1° rotations. Rotational errors also caused increased volumes of the OARs receiving the tolerance dose when the OARs were in close proximity to the target volumes. In these cases, it is likely that the OARs are located in or near the high dose gradient areas and a small rotational error would cause a significant dosimetric effort. Therefore, close attention should be paid to clinical cases exhibiting these characteristics.

In our study, systematic rotational setup errors were simulated by rotating the CT images and contours in the room coordinate system, which provided the most direct visualization of treatment geometries between the tumors and OARs. Since the commercial TPS did not provide the tools for image rotation and dose calculation on the rotated images, image and structure rotations were achieved using home‐developed software. Patient rotation can also be simulated by adjusting the treatment parameters. For example, a roll error can be simulated with a simple gantry rotation, and a yaw error can be simulated with a couch rotation.^(^ ^13^ ^,^ ^16^ ^,^ ^8^ ^)^ However, no simple method is available for pitch correction. Yue et al.^(9)^ developed mathematical formalisms that involved a combination of isocenter shift, and gantry, couch, and collimator rotations. Although the method can be used to correct patient setup errors in full six DOF, the derived combination of gantry, collimator, and couch rotations to simulate patient rotation is nonintuitive and is limited by mechanical limitations of machine movement and potential machine collision. Furthermore, it is difficult to apply to certain types of treatment (such as arc therapy) where gantry or collimator rotates continuously during beam on. Since the coordinate transformation is difficult to visualize, it is essential that phantom study with a set of fiducial markers be used to verify that the derived beams possess the same geometry relative to the target as in the plan. In contrast, the direct simulation method in our study provides the simple verification and spatial visualization for the patient and tumor OARs geometry.

The dosimetric effect of rotational setup errors would vary for different treatment sites and techniques. When tight margins are used and steep dose gradients are present, the chance of a geographic miss will be higher. For head‐and‐neck treatment, the body contour may change considerably from one point to another, and the targets are larger and irregular, which would make them more susceptible to rotational setup errors. Recent studies found that rotational errors contribute to treatment uncertainties for head‐and‐neck IMRT patients.^(^ ^21^ ^,^ ^22^ ^)^ Kim et al.^(13)^ studied the dose impact of head roll setup errors during head‐and‐neck IMRT treatments to the spinal cord and found an average increase in the maximal spinal cord dose of 3.1% and 6.4% for 3° and 5° angles of rotation, respectively. Sejpal et al.^(18)^ evaluated the dose changes in prostate cancer patients treated with proton therapy by rotating the gantry and couch to simulate the roll and yaw directions. They found no significant dose changes to targets and OARs when patient rotational errors were less than ± 5°. Fu et al.^(14)^ studied the dose delivery errors that would result from systematic rotational setup errors for prostate cancer patients receiving three‐phase sequential boost IMRT treatments. They reported that, for systematic rotational setup errors of up to 3°, the probability of dosimetric deviation in prostates larger than 2% is small. Slightly larger influences were observed for seminal vesicles. These studies provided general guidance for patient setup corrections for IGRT. However, each treatment site should be evaluated carefully considering the treatment techniques used and the tumor characteristics.

## V. CONCLUSIONS

We have evaluated the dose‐delivery errors resulting from systematic rotational setup errors for fractionated SRT using direct simulation in a TPS. We found that for a CTV‐to‐PTV margin of 3 mm, rotational setup errors of 3° or less did not decrease the CTV coverage of $V_{100\text{Rx}}$ to less than 95% in most cases. However, the target coverage decreased significantly for large targets with irregular or elliptical shapes when rotational errors of 5° or more were present. Noticeable increase in the volumes receiving tolerance doses were observed for OARs in close proximity to the target volumes. The results indicate that setup margins are warranted even in the absence of translational setup errors in order to account for rotational setup errors. Rotational setup errors should be evaluated carefully for clinical cases involving large tumor sizes and for targets with elliptical or irregular shape, as well as when the isocenter is away from the center of the PTV, or when OARs are in close proximity to the target volumes.
